# The Me and the Us of Emotions: a cluster-randomized controlled trial of the feasibility and efficacy of a compassion-based social–emotional learning program for children

**DOI:** 10.3389/fpsyg.2023.1196457

**Published:** 2023-11-01

**Authors:** Ana Xavier, Paula Vagos, Lara Palmeira, Paulo Menezes, Bruno Patrão, Sofia Abreu Mendes, Marta Tavares

**Affiliations:** ^1^Department of Psychology and Education, Portucalense University, Porto, Portugal; ^2^Center for Research in Neuropsychology and Cognitive and Behavioral Intervention (CINEICC), University of Coimbra, Coimbra, Portugal; ^3^William James Research Center, Departamento de Educação e Psicologia, Universidade de Aveiro, Aveiro, Portugal; ^4^Institute of Systems and Robotics (ISR), University of Coimbra, Coimbra, Portugal; ^5^Centro de Investigação em Psicologia para o Desenvolvimento, Instituto de Psicologia e Ciências da Educação, Universidade Lusíada Porto, Porto, Portugal; ^6^Agrupamento de Escolas de Valadares, Vila Nova de Gaia, Portugal

**Keywords:** social–emotional skills, compassion, clinical trial, children, school setting

## Abstract

There are well-established benefits of social and emotional learning (SEL) programs for children within educational contexts. Combining social–emotional skills and compassion abilities has been seldomly done, and it may be valuable at individual and societal levels, for resilient, empathetic, and inclusive societies. This study explored the feasibility and efficacy of a program designed to promote socioemotional and compassion skills in children attending the 3rd and 4th grades, by using in-class dynamics complemented with serious games. This program, named “The Me and the Us of Emotions,” is part of the Gulbenkian Knowledge Academies 2020 and consists of 10 group sessions embedded in the school curriculum. Using a cluster-randomized controlled trial design, school classes were allocated to intervention (classes, *n* = 8; children, *n* = 163) and control groups (classes, *n* = 6; children, *n* = 132). During the program, facilitators assessed adherence to the sessions’ plan, attendance, dosage (i.e., how many sessions were delivered), and participant responsiveness. Children completed self-report measures of social–emotional skills and emotional climate at pre-, post-intervention, 3-month, and 6-month follow-ups. Results indicate that the program is feasible, with high adherence, high attendance rate, and participant responsiveness. Results also indicate empathy, soothing, and drive feelings to change from pre-intervention to all other assessment moments, for the intervention group only. Moreover, cooperation and threat changed over time for participants in both the control and the intervention groups. The current study offers empirical support for the feasibility and utility of a compassion-based social–emotional learning program on promoting children’s empathy, and emotions of soothing and vitality in the school context. Thus, these findings contribute to recent research on the potential added value of compassion practices within an SEL program.

## 1. Introduction

During the last two decades, schools, families, researchers, practitioners, and policymakers have acknowledged the importance of promoting social and emotional skills in school contexts to foster children’s cognitive development, mental health, and well-being (Denham et al., 2009; Durlak et al., 2011; Elias et al., 2019; OECD, 2021a,b). These social–emotional competencies in early childhood have been found to be predictive of better academic achievements (Durlak et al., 2011; Domitrovich et al., 2017; Corcoran et al., 2018) and long-term life success (Clarke et al., 2015).

Social–emotional learning (SEL) programs aim to help individuals develop those skills, including self-awareness, self-management, social awareness, relationship skills, and responsible decision-making (Collaborative for Academic, Social and Emotional Learning, 2015). These programs are designed to be integrated into the curriculum of schools and are often offered to whole classes in their classrooms (Gueldner et al., 2020). SEL programs typically involve a combination of experiential strategies, such as role-playing, group discussions, and interactive activities. Regarding core components of SEL programs, identifying one’s own and others’ feelings is frequently addressed, before preparing children to learn behavioral coping skills (Lawson et al., 2019). Through systematic instruction, socioemotional skills may be taught, modeled, and practiced (Weissberg et al., 2015). The goal is to create a safe and supportive learning environment that allows students to explore and express their emotions, while also learning how to manage them effectively and use these skills as part of their daily repertoire of behaviors (Weissberg et al., 2015).

These positive school climates hold a dynamic interaction with student academic, personal, and social development (Coles, 2015; Berg et al., 2017; Elias et al., 2019). Caring and safeness environments facilitate students’ interactions with teachers and peers and provide positive conditions for learning from early childhood (Mondi et al., 2021) to adolescence (Coles, 2015; Osher et al., 2015; Berg et al., 2017). In contrast, threats to physical and psychological safety impair student’s emotional and behavioral functioning, as well as their attention and working memory, and can result in academic disengagement, school absenteeism and underachievement (Aronen et al., 2005; Berg et al., 2017; Elias et al., 2019; Cipriano et al., 2021). The training of emotional competencies, encompassing skills such as recognizing, expressing, and managing emotions, establishes a foundation for healthy development (Collaborative for Academic, Social and Emotional Learning, 2015; Berg et al., 2017). It empowers young people to effectively interact with others and navigate their surroundings, cope with stress, foster mental and emotional health, succeed academically, and thrive in both personal and academic realms (Osher et al., 2015). These socioemotional competencies are valuable in academic settings that require problem-solving, language and communication skills, collaboration and teamwork, and academic engagement and motivation. Thus, it is expected that grades and academic functioning (e.g., study skills, and on-task behavior) will also be positively impacted by SEL interventions (Cipriano et al., 2021).

A meta-analysis of universal school-based SEL programs, involving 213 schools from kindergarten through high school, showed that these programs can be effective in improving social and emotional skills, attitudes toward self and others, positive social behavior, and academic performance (e.g., reading or math achievement tests scores and grades), while also reducing conduct problems and internalizing symptoms (Durlak et al., 2011). Additionally, SEL programs have been found to improve students’ social skills and to prevent externalizing symptoms and risk behaviors (Sklad et al., 2012). Similarly, another meta-analysis (Taylor et al., 2017) involving 82 studies of SEL programs implemented from kindergarten through high school demonstrated benefits on students’ emotional skills, positive attitudes, prosocial behavior (e.g., cooperation), and academic performance (e.g., achievement test scores). Recently, a systematic review and meta-analysis including SEL intervention studies from 53 countries indicate benefits for students, namely increased socioemotional skills, civic attitudes, prosocial behaviors, school functioning, and diminished externalizing behaviors and emotional distress, with large effect sizes; these benefits were found for the whole sample, and so were not particular to any cultural context (Cipriano et al., 2021). SEL programs may additionally impact on scholastic performance. A meta-analysis (Corcoran et al., 2018) indicated that SEL interventions generally have a positive effect on reading and mathematics performance and a smaller effect on science achievement. Also, students who benefit from SEL demonstrated improvement in academic achievement, with a medium effect size (Cipriano et al., 2021).

When focusing on elementary schools, Jones et al. (2017) reviewed 11 SEL programs RCT studies and pointed out ambiguous results, which may be related to the measures that were used. Alternatively, other works indicate the efficacy of SEL programs through RCT in elementary schools (from third to fifth grade). Such gains have also been observed for elementary SEL intervention, with students improving their proficiency in reading, writing and math (Schonfeld et al., 2015). Particularly, the MindUP revealed that children improved in empathy and perspective-taking, and decreased depression symptoms and peer-rated aggression, with moderate effects (Schonert-Reichl et al., 2015). The PATHS shows small effects on social problem-solving (e.g., aggression, externalizing problems), and the reduction of aggressive behavior tends to occur only during the second year of implementation (Crean and Johnson, 2013).

Still, studies investigating the stability of gains in follow-up moments are scarce. One of them found reduced effects of changes after 6 months (Durlak et al., 2011). One meta-analysis conducted by Taylor et al. (2017), which included SEL studies with follow-up periods ranging from 6 months to 18 years, indicated maintained gains with modest effect sizes. Regarding follow-up studies, 11% of studies considered by Cipriano et al. (2021) in their systematic review and meta-analysis did a 6-month assessment after the end of the intervention and results indicated maintained improvements in socioemotional skills, and reductions in externalizing behaviors and emotional distress, with an exception for prosocial behaviors and school functioning (including academic achievement, study skills, and academic performance). Further studies are encouraged and advised to include an assessment of follow-up and quality of implementation (Durlak et al., 2011; Durlak, 2016; Taylor et al., 2017; Gadke et al., 2021).

Another way of framing the ability to manage one’s emotions is based on the development of compassion skills. Compassion-based programs for children are designed to help young people develop empathy and understanding towards others, as well as to learn how to manage their own emotions. From an evolutive perspective, humans are inherently social, with the capacity for perspective-taking, compassion, empathy, love, and altruistic behavior (Gilbert, 2005; Szalavitz and Perry, 2010). These social skills strengthen the connection and attachment within groups. Early warmth and safeness experiences and the continuity of nurturing environments among educational and societal systems are needed to reinforce the human biological ability to be compassionate (Coles, 2015). According to Compassion-Focused Therapy (CFT; Gilbert, 2009, 2014), human motivations to explore the world, compete for resources, and belong to a group are linked to emotions that guide our behaviors. These emotions are grouped into three basic emotion-regulation systems oriented for adaptive functions, namely self-protection and survival (Gilbert, 2009, 2014). The threat system is linked to emotions like fear, anger, and disgust, thus helping us quickly identify and respond to threats. The drive-excitement system is linked to emotions of excitement and joy, which motivate and energize us to explore and pursue resources. The soothing-affiliative system is linked to feelings of calmness, contentment, and safeness, and orientates us to give and receive care from others (Gilbert, 2009, 2014). This soothing system plays a crucial role in regulating distress and feeling socially safe and connected (Kirby et al., 2017a). Compassion is rooted in this affiliative system and is defined as a “sensitivity to suffering in self and others with a commitment to try to alleviate and prevent it” (Gilbert and Choden, 2013, p. 94). In fact, empathy and compassion are important components of prosocial behavior (e.g., helping, caring, sharing; Chierchia and Singer, 2016), which can lead to greater peer acceptance and positive interactions in children and adolescents (Cheang et al., 2019). Compassion-based approaches can be effective in producing changes in cooperation, trust, and tolerance (Chierchia and Singer, 2016).

In compassion-based approaches, participants learn how to activate their soothing system, through mindfulness, loving-kindness and compassion meditations, imagery practices, and compassionate letter writing (Gilbert, 2009; Gilbert and Choden, 2013; Neff and Germer, 2013; Gilbert, 2014). The benefits of these practices in a daily base routine include increased self-compassion (Kirschner et al., 2019), empathy or warmth toward others (Klimecki et al., 2014), and social connectedness (Hutcherson et al., 2008). Despite robust evidence of the benefits of compassion-based interventions on well-being in adults (Kirby et al., 2017b), data is only preliminary in young people. For instance, in the *Making Friends with Yourself: A Mindful Self-Compassion Program for Teens* (Bluth et al., 2016), adolescents reported higher levels of self-compassion, life satisfaction, and lower levels of depression after the intervention, in comparison with a waiting list group. Additionally, an online four-week Self-Compassion Program showed that children aged 8–11 years old reported greater self-compassion, positive emotions, and lesser anxiety at the end of the program (Karakasidou et al., 2021). These findings suggest not only that self-compassion training may be applied to younger populations but also encourage universal actions targeting compassion in schools.

Based on the previous evidence on SEL programs and compassion-based interventions, both approaches share similar components, namely the promotion of self-awareness (including emotion identification) and emotional regulation (i.e., coping with difficult emotions), social awareness (including empathy and compassion), and relationship skills (including cooperation, helping, sharing). Despite their shared components, both approaches have not been used complementarily. Thus, the continuous development and implementation of these approaches in a complementing way, for children in school contexts, is still needed to foster positive and cooperative school environments (Coles, 2015; Welford and Langmead, 2015; Elias et al., 2019; OECD, 2021b). Also, there seems to be a paucity of research focusing on the SEL and compassion or kindness programs for children in the first school years, although literature suggests SEL training in early childhood may benefit healthy development (Mondi et al., 2021).

In addition to this complementary approach, a few works have recently integrated serious games as a complement to SEL programs, particularly to promote social skills (Girard et al., 2013; Zheng et al., 2021). Serious games are video games used for educational purposes (e.g., training, knowledge acquisition, skills development; Girard et al., 2013). In this context, serious games help to create an interactive and appealing educational environment, which benefits children by improving their cognitive abilities and positive attitudes toward learning (Lamb et al., 2018; Zhonggen, 2019). Zheng et al. (2021) showed that serious games used as an activity for promoting SEL components (mainly social skills) seem useful when paired with in-person guided discussion. Additionally, the use of serious games seems to be well-accepted as a part of SEL programs. In fact, recent results suggest that children’s enjoyment and interest in the subjects addressed in a SEL program was partially explained by the use of serious games (Xavier et al., 2022).

Based on the theoretical principles outlined above concerning the SEL and compassion-focused therapy we design The Me and the Us of Emotion. It was developed based on a complementary approach to those two theoretical frameworks, and resorts to serious games in addition to in-person dynamics. The Me and the Us of Emotions is a universal school program integrated into the Gulbenkian Knowledge Academies 2020; for further detail, please see section 2.4 below. The current study aims to analyze the feasibility and efficacy of The Me and the Us of Emotions, using a cluster-randomized controlled design. These findings may contribute to the widening of empirically supported programs applied in ecological settings that aim to promote emotional well-being in children.

Regarding feasibility, we considered indicators of adherence, attendance, dosage, and participant responsiveness. These indicators were chosen based on Carroll et al.’s (2007), Durlak’s (2016), and Gadke et al. (2021) recommendations for good practices in program implementation. As for efficacy, we investigated changes in outcome variables at pre-, post-intervention, 3 and 6-month follow-ups between two groups (one intervention group and one control group). We addressed outcome variables directly related to the goals of The Me and the Us of Emotions. Specifically, we considered three domains of socioemotional skills (i.e., emotional control, empathy, and cooperation) in relation to the programs’ intent of promoting skills related to emotion identification, and to understand others’ feelings and perspectives. Additionally, we assessed the perception of emotional climate based on the three emotional regulation systems (threat, soothing and drive feelings) because the program addresses emotion regulation skills oriented toward self-reassuring and self-compassion. Based on the previous evidence on SEL programs producing positive effects on social–emotional outcomes (Schonert-Reichl et al., 2015; Jones et al., 2017), we hypothesize that the intervention group, compared with the control group, will display more empathy and cooperation skills, and perceive a more positive emotional climate (particularly, soothing, and drive feelings) throughout the assessment moments.

## 2. Methods

### 2.1. Study design

This study is a cluster-randomized controlled trial (cluster RCT) with a random allocation at the school class level. A member of the research team did the random allocation using a computer-based random allocation, and eligible classes were randomly assigned to an intervention or to a control group condition, with a proportion of 43 and 57%, respectively.

### 2.2. Participants’ recruitment and characterization

**Figure 1:**
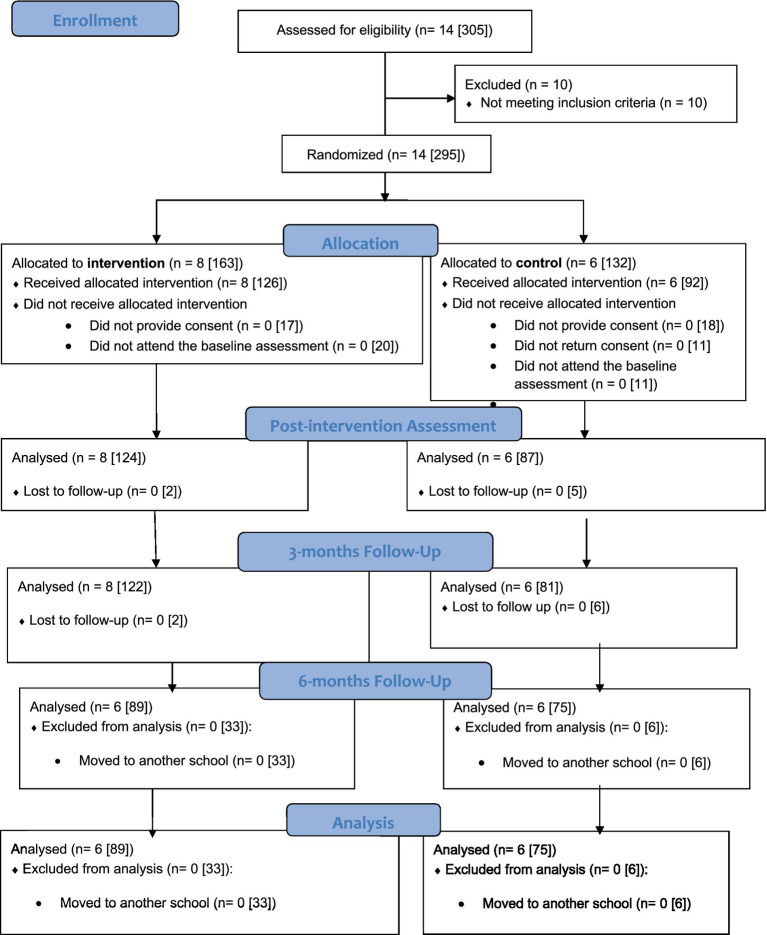
The consort flow diagram. Flow of school-classes and children through the study. All numbers are classes [children].

Figure 1 displays the flow of the recruitment process, considering school classes and participants. At the school class level, inclusion criteria were: (a) classes from 3rd and 4th grades, and (b) classes that had no previous SEL intervention. Furthermore, at the participant level, the inclusion criterium referred to (c) children with no disability that might be an impediment to answering the data collection questionnaires. Thus, children with disabilities were excluded prior to randomization but no classes were excluded for this reason. During the session, these children were given another task by the teacher. A total of 305 children fulfilled these criteria across two public schools located in the same geographical area, for a total of 14 classes. Those children were invited to take part in this research and parental consent was asked. We received parent informed consent from 249 children (*n* = 35 children with no informed consent). Another thirty-one children failed to attend the baseline assessment. After baseline assessment, fourteen school classes were randomized to the intervention group (*N* = 8) or the control group (*N* = 6).

For this study, only data for children who completed all assessment moments was used for the intervention group and the control group. Thus, the final sample was comprised by 164 children aged between 8 and 10 years old (i.e., *n* = 89 for the intervention group and *n* = 75 for the control group). Table 1 presents the sociodemographic characteristics of the final sample. There are no differences in age between groups, *t*_(151.206)_ = −1.33, *p* = 0.186, and no differences in the prevalence of boys and girls across the intervention and control groups, *X*^2^_(1)_ = 0.96, *p* = 0.326. The distribution of years of education between groups is marginally significant, *X*^2^_(1)_ = 3.84, *p* = 0.050. Urban and rural residence distribution is similar for both groups, *X*^2^_(1)_ = 0.17, *p* = 0.679.

**Table 1:** Sociodemographic characteristics of participants at 6-month follow-up.

	Intervention group (*n* = 89)	Control group (*n* = 75)	Total sample (*n* = 164)
**Gender [*n* (%)]**			
Male	43 (48.3)	42 (56)	85 (51.8)
Female	46 (51.7)	33 (44)	79 (48.2)
**Years of education [*n* (%)]**			
3rd Grade	59 (66.3)	60 (80)	119 (72.6)
4th Grade	30 (33.7)	15 (20)	45 (27.4)
**Residence [*n* (%)]**			
Urban	58 (86.6)	36 (83.7)	94 (14.5)
Rural	9 (13.4)	7 (16.3)	16 (85.5)
Age [M (SD)]	8.87 (0.66)	8.72 (0.73)	8.80 (0.69)

### 2.3. Procedure

Ethical approvals were obtained from the Ethical Committee for Health of the Higher Education institution hosting this research project, after which two public schools in the northern region of Portugal were contacted and asked for collaboration to recruit participants and implement the intervention in the school context. The study was conducted according to the guidelines of the Declaration of Helsinki. Parents or legal guardians were informed about the study’s aims and procedures by e-mail and video and gave their informed written consent for data collection. Children enrolled in the study were fully informed about the study’s goals and the aspects of confidentiality, and then also gave their written consent to take part of this study. They agreed to voluntarily participate and fill out the instruments in the classroom in the presence of the teacher and of at least one research team member. When necessary, clarification regarding the self-report protocol was provided. The intervention program occurred between January and April 2021 (i.e., between the pre-intervention [T1] and post-intervention [T2] data collection). The 3-month [T3] and 6-month [T4] follow-ups occurred in July and October 2021, respectively.

### 2.4. Description of The Me and the Us of Emotions program

The Me and the Us of Emotions is a universal program based on the SEL framework (Collaborative for Academic, Social and Emotional Learning, 2015; Gueldner et al., 2020), and compassion-based approaches (Gilbert, 2009, 2014; Bluth, 2017). It was developed by the research team for children in the 3^rd^ and 4^th^ grades and comprises ten manualized developmentally appropriate weekly group sessions, with a duration of 60 min each, to be run in the classroom in the presence of the teacher. Four psychologists with previous training in the program and one clinical psychology master student delivered the group sessions (two facilitators per class). The program was developed to be preferably provided in person but can also be applied online, if necessary, with all activities having been adapted to the online format. Because this study was conducted during the COVID-19 pandemic global crisis, the baseline assessment, the first session and the final four sessions were conducted in person whereas sessions 2 to 6 were delivered online through videoconference.

Table 2 displays a session-by-session overview of the program. The program included the following main components: (a) psychoeducation regarding the universality and adaptive function of emotions; (b) psychoeducation on the different physiological, cognitive, and behavioral components of basic emotions (e.g., joy, sadness, fear, anger) and secondary emotions (e.g., self-reassuring and compassion); (c) self-reassuring and self-compassion exercises to tackle difficult emotions and to enhance children’s ability to be kind to themselves; and (d) compassion and cooperative actions to promote collaborative and prosocial behaviors. Experiential exercises and key messages were developmentally adapted or based on pre-existent social–emotional practices with children (e.g., turtle exercise from Webster-Stratton, 1999) and compassion-based approaches (e.g., compassionate touch exercise from Bluth, 2017; safe place meditation and compassionate letter adapted from CFT; Gilbert, 2009). The rationale underlying all sessions is that the practice of socioemotional skills oriented towards self-reassurance and cooperation can be modelled, learned, and practiced, through explicit instruction and/or continuous encouragement (Gilbert, 2009, 2014), and consequently be applied to diverse situations in day-to-day life (Durlak et al., 2011; Weissberg et al., 2015).

**Table 2:** Overview of The Me and the Us of Emotions program session-by-session.

Sessions		Aims	Exercises and practices
1	What are emotions	Establish the group rules and presentation of the participants; Understand the importance and adaptive function of emotions, as well as the universality of emotions.	Group activity of self-presentation promoting shared human condition.
2	Joy	Psychoeducation about the joy emotion on its physiological, cognitive, and behavioral components. Acknowledgment and identification of own’s joy and others’ joy and behaviors.	Exercise of the recipe of joy. Serious game. Inter-session activities for the classroom (work on identifying the emotion of joy) and home (creating the recipe for family joy).
3	Self-reassuring	Psychoeducation about the self-reassuring emotion on its physiological, cognitive, and behavioral components. Familiarization and training in strategies to promote the emotion of safety/reassurance.	Safe place visualization exercise. Inter-session activities for the classroom (safe place draw) and home (safe family place music).
4	Self-compassion	Acknowledgment and identification of difficult emotions in face of failure and setbacks. Familiarization and training in strategies to promote the emotion of self-compassion.	Exercise of the compassionate touch. Hand drawing exercise with self-compassionate phrases. Inter-session activities for the classroom (exhibition with compassionate phrases) and home (practice the exercise of compassionate touch).
5	Empathy and perspective taking	Promote perspective-taking and empathy skills. Promote the skills of understanding what the other is feeling and knowing how to read the other’s emotions.	Exercise of multiple perspectives. Inter-session activities for the classroom and home (stimulating the discovery of multiple perspectives in different contexts).
6	Compassion for Others	Promote cooperative and compassionate behaviors toward others (e.g., offer help, support, and understand). Encourage prosocial behaviors to help alleviate the suffering of the other (e.g., emotionally encourage, hug).	Visual and auditory exercise through video watching of the “hugs song” (by Godinho, 1988). Hangman game. Inter-session activities for the classroom and home (Calendar of Compassionate Actions).
7	Sadness	Psychoeducation about the sadness emotion on its physiological, cognitive, and behavioral components. Promote the normalization of the sadness emotion and the adoption of adaptive strategies to make the sadness emotion less difficult.	Serious game. Inter-session activities for the classroom (designing posters with strategies for dealing with sadness emotion) and home (emotion drawing).
8	Fear	Psychoeducation about the fear emotion on its physiological, cognitive, and behavioral components. Promote the normalization of fear emotion (understand its protective function versus impairment symptoms). Promote the use of strategies to ask for help and to approach unpleasant emotion.	Serious game. Inter-session activities for the classroom (work referring to “true fears and lying fears”) and home (interviewing others about their fears).
9	Anger	Psychoeducation about the anger emotion on its physiological, cognitive, and behavioral components. Understand the adaptive function of anger versus externalizing behaviors as disruptive. Familiarization and training in strategies to promote anger regulation.	Turtle technique exercise. Serious game. Inter-session activities for the classroom and home (practicing and teaching the turtle technique).
10	Emotions for Life	Identification of the diverse emotions taggled in the program. Identification and reflection about the gains with the program. Promote the anticipation of circumstances that are likely to provoke unpleasant emotions in the future and the strategies to effectively deal with it.	Serious game. Activities for the classroom (work group about one of the emotions discussed throughout the program and compassionate letter writing for one character of the program) and at home (Compassionate Letter Writing).

The sessions include several action strategies: psychoeducation based on images and videos expressing the daily experiences of two characters built for this intervention; guided reflection and discussion, involving reflection/brainstorming of ideas guided by the facilitator, constructive feedback, and positive reinforcement; active engagement, *via* manual and experiential activities (e.g., exercises in imagery), and the use of serious games addressing the contents of each session. Several sessions (*cf.* Table 2) are supplemented with serious games that use the session’s theme to promote emotional identification and effective strategies for dealing with difficult emotions (e.g., sadness, fear, anger). In addition, serious games can be accessed from home and between sessions from the project’s web platform.^1^ Each session includes suggested activities for the classroom and home between sessions and for the end of the program (e.g., in the fear session it is suggested drawing activities about real and unreal fears to be exposed in the classroom and interviewing parents about their own fears). These activities were suggested to the class (including teachers and children) at the end of each session. Teachers, as passive observers of the sessions, were invited to remind and encourage children to complete inter-session activities and to take part of a final group-class activity scheduled for after the end of the program implementation.

### 2.5. Instruments

#### 2.5.1. Demographic characteristics

Sociodemographic data were collected regarding gender, age, academic year, and residence (urban or rural). This information was used the characterize our groups (see above).

### 2.6. Feasibility measures

The systematic assessment of the intervention implementation was encouraged by the Gulbenkian Knowledge Academies 2020 through periodic meetings and training for the Academies. To guarantee the fidelity of the intervention implementation, the research team provided training (40 h training, with 12 h happening before the beginning of the intervention) and supervision (1 h weekly session) to the facilitators. The research team developed and provided a guided and structured manual that organized and detailed each sessions’ plans and explained each activity. Also, all material and digital supports (e.g., power-point of each session, worksheets, serious games) were provided. A monitoring sheet was also provided for the facilitators to complete at the end of each session. Facilitators reported the level of adherence to the session plans, the number of students that attended each session, and the participant’s responsiveness. These elements were informative on the feasibility and fidelity of the program implementation, according to Carroll et al.’s (2007), Durlak’s (2016), and Gadke et al. (2021) recommendations.

#### 2.6.1. Adherence

This was assessed by the facilitators of the program regarding whether the program was being implemented as it was originally designed. For each session, the facilitator assessed “*how close to the original plan do you think this session was developed in this group?*” according to a 5-point response scale ranging from 1 = *very little* to 5 = *totally*.

#### 2.6.2. Attendance/dosage

This was assessed by the facilitators of the program through the completion of an assiduity sheet, as a measure of how many sessions each participant received.

#### 2.6.3. Participant responsiveness

This was assessed by the facilitators of the program regarding how participants were engaged, involved, or responded to the program (Carroll et al., 2007). Four questions were used: “*How involved were the children in the interactive game?*”; “*How involved in this session do you think the students were?*”; “*How well do you think the students behaved, according to the rules, during the session?*”; “*How supportive and committed to the program was the teacher during the session?*.” The facilitators answered each question using a 5-point Likert scale ranging from 1 = *very little* to 5 = *very much*.

### 2.7. Outcomes measures

Study on social and emotional skills (SSES; OECD, 2019) is a large-scale international survey assessing children and adolescents’ social and emotional skills. The Portuguese adaptation is ongoing by the monitoring and assessment team of the Gulbenkian Knowledge Academies 2020. In the present study, three subscales were chosen based on the target age group and on the aims of the intervention. These subscales assessed (i) emotional control (8 items, e.g., “*I keep my emotions under control*”), (ii) empathy (8 items, e.g., “*I care about what happens to others*.”), and (iii) cooperation (8 items, e.g., “*I like to help others*”). The participant is asked to answer about the agreement with each item on a 5-point Likert scale, ranging from *strongly disagree* (1) to *strongly agree* (5). Some items are reversed scored. Higher scores indicate higher levels of emotional control, empathy, and cooperation, respectively. In the original version, Cronbach’s alphas were 0.74 for emotional control, 0.66 for empathy, and 0.80 for cooperation. In the present study, Cronbach’s alphas for pre-intervention were α ≥ 0.71 for emotion control, α ≥ 0.69 for empathy, α ≥ 0.82 for cooperation. For post-intervention, Cronbach’s alphas were 0.77, 0.79, and 0.85 for emotional control, empathy, and cooperation subscales, respectively. For the 3-month follow-up, Cronbach’s alphas were. 78, 0.77, and 0.85 for emotional control, empathy, and cooperation subscales, respectively. Finally, for the 6-month follow-up, Cronbach’s alphas were.82, 0.82, and.86 for emotional control, empathy, and cooperation subscales, respectively.

Emotional climate in the classroom scale – children (ECCS – C; Albuquerque et al., 2019). This self-report measure was developed based on the affect regulation systems model proposed by Gilbert (2009, 2014) and assesses the presence/activation of those three systems, namely threat, drive, and soothing/safeness. This scale was developed for 8 to 12-year-old children using a focus group to improve the readability and clarity of the items’ content. It asks children about how they feel in the classroom and to complete 15 items about emotions (such as anger, calm, and active), which represent the three subscales (five items for each type of emotion system). Higher scores indicate higher levels of threat, drive, or soothing/safeness emotions perceived in the classroom. The applicability of this scale to children is under study, but two previous studies with a similar scale for children and adolescents show adequate internal reliability of the measure (Gonçalves, 2019; Henriques, 2019). In the current study, Cronbach’s alphas for the combined intervention and control groups at pre-, post-intervention, 3 and 6-month follow-up were 0.64, 0.68, 0.64, 0.65 for threat subscale, 0.71, 0.72, 0.78, 0.80 for soothing subscale, and 0.80, 0.80, 0.83, 0.87 for drive subscale.

### 2.8. Data analysis

All data analyses were performed using IBM SPSS Statistics for Mac (Version 27.0). The assumption of multivariate normality was analyzed for outcome variables (i.e., emotional control, empathy, cooperation, threat, soothing, and drive) in all four assessment moments and there was no severe violation of normal distribution (|Sk| < 3 and |Ku| < 8–10; Kline, 2015). To compare groups’ scores in our outcome measures across four assessment moments, we performed 2 (condition) × 4 (time) mixed-model ANOVAs to analyze the between-subjects effect of group (intervention group vs. control group), the within-subjects effect of time (pre-intervention, post-intervention, 3-month, and 6-month follow-up), and interaction effects. Levene’s test for homogeneity of variances between groups was analyzed. The sphericity assumption for the repeated measures ANOVAs was analyzed through Mauchly’s W test. Whenever this assumption was not verified, the Greenhouse–Geisser epsilon (ε < 0.75) was used, which corresponds to a probability correction factor of the F-statistics’ significance by adjusting the degrees of freedom (Field, 2013). Eta partial squared was used as a measure of effect size and was interpreted as follows: >0.14 indicates a large effect; >0.06, a medium effect, and > 0.01, a small effect (Cohen, 1992). *Post-hoc* tests using Bonferroni were analyzed for pairwise comparisons.

Missing values were not missing completely at random for items of the Social and Emotional Skills across the four assessment moments for the intervention and control groups, Little MCAR = *X^2^*_(2488)_ = 3888.08, *p* < 0.001. Similarly, data were not missing completely at random for items of the Perceived Emotional Climate in Classroom Scale for the intervention and control groups across four assessment moments, Little MCAR *X^2^*_(2193)_ = 2604.42, *p* < 0.001. Incomplete data represented 0.59 and 0.79% of the possible data pool and affected 22.28 and 27.16% of participants, respectively. To avoid sample loss, a pairwise deletion approach was used to each individual measure under scrutiny.

## 3. Results

### 3.1. The Me and the Us of Emotions’ feasibility

#### 3.1.1. Adherence

Results show high levels of adherence (*Min* = 3, *Max* = 5, *M* = 4.81, *SD* = 0.46), meaning that all sessions were at least adequately implementer (i.e., minimum response value of 3), and that, on average, sessions were implemented very close to totally as planned.

#### 3.1.2. Attendance/dosage

All 10 sessions were delivered, and the intervention had a high attendance rate, ranging between 81% in session 6 and 96% in session 8.

#### 3.1.3. Participant responsiveness

Results indicate high levels of engagement in serious games (*Min* = 3, *Max* = 5, *M* = 4.78, *SD* = 0.58) and in the sessions (*Min* = 4, *Max* = 5, *M* = 4.83, *SD* = 0.37). In addition, facilitators indicate that children behaved well in the sessions (*Min* = 2, *Max* = 5, *M* = 4.72, *SD* = 0.60), and teachers, as observers, were highly supportive and committed during the sessions (*Min* = 3, *Max* = 5, *M* = 4.76, *SD* = 0.49).

### 3.2. Changes in outcome measures, across groups and assessment moments

Descriptive values for all measures across four assessment moments are presented in Table 3, for the complete sample and across groups.

**Table 3:** Descriptive values for outcome measures at pre-intervention and post-intervention for the total sample, the intervention group, and the control group.

	Total sample				Intervention group				Control group			
	Pre	Post	3-month	6-month	Pre	Post	3-month	6-month	Pre	Post	3-month	6-month
**Social-emotional skills**												
Emotional control	3.46 (0.73)	3.42 (0.81)	3.40 (0.81)	3.46 (0.80)	3.36 (0.68)	3.39 (0.75)	3.37 (0.71)	3.43 (0.69)	3.59 (0.78)	3.47 (0.89)	3.44 (0.94)	3.49 (0.90)
Empathy	3.82 (0.60)	3.99 (0.67)	3.97 (0.66)	3.92 (0.69)	3.79 (0.53)	4.05 (0.62)	3.99 (0.66)	4.01 (0.62)	3.82 (0.69)	3.91 (0.73)	3.93 (0.67)	3.83 (0.76)
Cooperation	4.23 (0.59)	4.33 (0.63)	4.32 (0.61)	4.36 (0.59)	4.23 (0.55)	4.37 (0.56)	4.37 (0.52)	4.37 (0.56)	4.23 (0.65)	4.28 (0.72)	4.25 (0.71)	4.34 (0.63)
**Emotional climate**												
Threat	1.79 (0.69)	1.96 (0.74)	1.90 (0.67)	1.77 (0.61)	1.84 (0.71)	2.04 (0.78)	1.92 (0.66)	1.83 (0.59)	1.73 (0.66)	1.84 (0.66)	1.86 (0.69)	1.71 (0.62)
Soothing	4.08 (0.78)	4.31 (0.66)	4.31 (0.73)	4.30 (0.71)	4.02 (0.75)	4.29 (0.64)	4.31 (0.73)	4.33 (0.63)	4.16 (0.82)	4.33 (0.69)	4.31 (0.73)	4.28 (0.79)
Drive	3.98 (0.89)	4.25 (0.74)	4.19 (0.76)	4.22 (0.79)	3.94 (0.84)	4.27 (0.72)	4.25 (0.76)	4.24 (0.79)	4.04 (0.96)	4.23 (0.77)	4.11 (0.76)	4.20 (0.79)

Values are presented as M (SD).

### 3.3. Change in social–emotional skills

Results for emotional control showed that the main effects of time and group were non-significant, respectively *F*_(2.74,404.86)_ = 0.80, *p* = 0.49; *F*_(1,148)_ = 1.61, *p* = 0.21. Similarly, the interaction effect was not statistically significant, *F*_(2.74, 404.86)_ = 0.56, *p* = 0.63.

For empathy, results showed a significant main effect of time, *F*_(2.81, 398.69)_ = 6.99, *p* < 0.001, *η*_p_^2^ = 0.047, with a medium effect size. Both the main effect of group, *F*_(1, 142)_ = 1.24, *p* = 0.27, and the interaction effect were not statistically significant, *F*_(2.81, 398.69)_ = 1.03, *p* = 0.38. Pairwise comparisons indicated significant differences for the intervention group only (and not for the control group, all *p*s > 0.49). Differences were located from pre-intervention in relation to all other assessment moments (i.e., *p* = 005 for post-intervention, *p* = 001 for 3-month follow-up, and *p* = 0.023 for 6-month follow-up). Participants in the intervention group reported more empathy at post-intervention and follow-ups, in comparison with the pre-intervention assessment (*cf.* Table 3).

For cooperation, results indicated a significant main effect of time, *F*_(3,453)_ = 3.55, *p* = 0.015, *η*_p_^2^ = 0.023, with a medium effect size. Both the main effect of group, *F*_(1, 151)_ = 0.88, *p* = 0.35, and the interaction effect of time and group were not statistically significant, *F*_(3,453)_ = 1.59, *p* = 0.197. On average, all participants reported more cooperation from pre-intervention to 6-month follow-up (*p* = 0.034) (*cf.* Table 3).

### 3.4. Children’s perception of emotional climate

Results indicated a main effect of time, *F*_(3,150)_ = 4.21, *p* = 0.006, *η*_p_^2^ = 0.027, for the threat system with medium effect sizes. The main effect of group, *F*_(3,150)_ = 0.75, *p* = 0.39, and the interaction effect were not statistically significant, *F*_(3,150)_ = 0.32, *p* = 0.81. On average, all participants reported perceiving significantly less threat from post-intervention to six-month follow-up (*p* = 0.027) (*cf.* Table 3).

For the soothing system, results showed a significant main effect of time, *F*_(2.57, 364.21)_ = 10.44, *p* < 0.001, *η*_p_^2^ = 0.068, with a medium effect size. No significant main effect of group, *F*_(1,142)_ = 0.25, *p* = 0.62, and no significant interaction effect were found, *F*_(2.57, 364.21)_ = 0.56, *p* = 0.61. Pairwise comparisons indicated significant differences for the intervention group only (and not for the control group, all *p*s > 0.10). Differences were located from pre-intervention in relation to all other assessment moments (i.e., *p* = 007 for post-intervention, *p* = 002 for 3-month follow-up, and *p* = 0.007 for 6-month follow-up). Participants in the intervention group perceived more soothing in their classrooms at post-intervention and follow-ups, in comparison with the pre-intervention assessment (*cf.* Table 3).

For the drive system, results showed a significant main effect of time, *F*_(2.55,379.84)_ = 29.64, *p* < 0.001, *η*_p_^2^ = 0.061, with a medium effect size. Both the main effect of group, *F*_(1,149)_ = 0.20, *p* = 0.65, and the interaction effect were not statistically significant, *F*_(2.55,379.84)_ = 1.25, *p* = 0.29. Pairwise comparisons indicated significant differences for the intervention group only (and not for the control group, all *p*s > 0.20). Differences were located from pre-intervention in relation to all other assessment moments (i.e., *p* = 003 for post-intervention, *p* = 003 for 3-month follow-up, and *p* < 0.001 for 6-month follow-up). Participants in the intervention group perceived more drive in their classrooms at post-intervention and follow-ups, in comparison with the pre-intervention assessment (*cf.* Table 3).

## 4. Discussion

Schools are primordial contexts to promote not only academic learning but also social–emotional skills in children and adolescents (OECD, 2021a,b). Social and emotional skills, such as understanding and managing emotions, dealing with social conflicts effectively, and making responsible decisions, have been shown to influence intra and inter-personal outcomes, namely improved emotional skills, positive attitudes, prosocial behavior, and academic performance, and reduced externalizing and risk behaviors (Durlak et al., 2011; Sklad et al., 2012; Schonert-Reichl et al., 2015; Jones et al., 2017; Taylor et al., 2017). In Portugal, the Gulbenkian Knowledge Academies (Calouste Gulbenkian Foundation, 2023) has been supporting projects aimed to promote adaptability, critical thinking, resilience, creativity, problem-solving, self-regulation, and communication for children and adolescents at diverse institutions (e.g., schools, and local associations). The Me and the Us of Emotions’ program falls within those projects and refers to a universal program developed to foster the capacity for emotion recognition; emotional self-regulation focused on reassurance and compassion; and behaviors of social connection and cooperation in children. This intervention was framed within the SEL and compassion-focused theoretical principles, being highly experiential and complemented with the use of serious games. The current study aimed to analyze the feasibility and efficacy of The Me and the Us of Emotions program on socio-emotional skills and children’s perception of emotional climate, using a cluster-randomized controlled design.

Regarding the feasibility indicators, we intended to contribute to the assessment of the quality of the program’s implementation, which is assumed as an essential component related to positive outcomes (Durlak et al., 2011; Durlak, 2016). Still, only a few studies examine the degree to which the program is implemented as planned, even if adherence and participant responsiveness may be predictors of participants’ SEL outcomes (Vroom et al., 2019). In addition, recent guidelines for feasibility studies postulate the importance of the acceptability of programs by the target population (Gadke et al., 2021). About The Me and the Us of Emotions, facilitators reported high adherence to the structured plan of the sessions. This may reflect not only an appropriate process of program development that resulted in an easily applied program but also the closeness and continuous monitoring and supervision provided by the research team. The program had a high attendance rate through all 10 sessions. The facilitators assessed the participants’ responsiveness with high engagement both in serious games and in the sessions. Additionally, facilitators considered that children behaved well in the sessions, and teachers, as observers, were supportive and committed during the sessions. Taken together, these findings suggest that The Me and the Us of Emotions is a feasible intervention for children and deliverable within the school context.

Regarding changes between groups across assessment moments, we found significant effects of time for social–emotional skills, particularly empathy. Specifically, only children in the intervention group reported increased levels of empathy from pre-intervention to all other assessment moments. Self-reported empathy seemed to have remained stable after the intervention. These results seem to be aligned with the contents of the program that emphasize that all emotions, even the undesirable ones, are helpful for our survival and self-protection and that emotional experience is not our fault and we do not control it, nor do we need to. These findings also align with previous studies focused on follow-up SEL programs, which showed modest results in improving social–emotional skills (Jones et al., 2017; Taylor et al., 2017), despite the well-known benefits for students and educational settings (Durlak et al., 2011; Sklad et al., 2012; Domitrovich et al., 2017; Corcoran et al., 2018). Still, about cooperation, a significant effect of time showed that both the intervention and the control group reported an average increase from pre-intervention to follow-up, which may be associated with the progressing of the school year, as it provides more opportunities for this kind of interaction to occur for all students. This result also aligns with previous findings showing that change in cooperation is more difficult to observe and be maintained over time, specifically following an intervention (Singer and Steinbeis, 2009; Crean and Johnson, 2013).

About the emotion-regulation systems from pre- to post-intervention, exploring the significant effect of time showed that emotions of soothing and drive valences increased from pre- to post-intervention and remained stable at both follow-up assessments, only for the intervention group. Given that the program’s sessions addressed basic emotions included in the emotion-regulation systems from CFT (Gilbert, 2009, 2014), namely, joy, sadness, fear, and anger, these results may reflect an increased awareness that children may have acquired about their own emotional experience. This result is in line with previous findings on compassion-based approaches promoting emotional well-being in children and adolescents (Bluth et al., 2016; Karakasidou et al., 2021). On the other hand, threat emotions decreased in both groups from the post-intervention to the 6-month follow-up assessment. It may be the case that students felt more pressure in the middle of the school year, which dissipated over time, particularly if we consider that summer vacation took place between these assessment moments.

### 4.1. Limitations and future directions

Though current findings are encouraging and relied on sound and highly replicable design, they should be considered within some limitations to be addressed in future studies. Since our sample came from two public schools in the same geographic region, this may limit the generalization of our results. The fidelity indicators we currently used are considered good practice in SEL programs (Carroll et al., 2007; Durlak, 2016), but they were not exhaustive. Although the adherence, attendance/dosage, and participant’s responsiveness were assessed, the differentiation and quality of delivery (i.e., what makes a program unique and how the facilitator coach, acts, and models with attitude and enthusiasm the socio-emotional skills; Carroll et al., 2007; Gadke et al., 2021) was not assessed due to the social restrictions arising from the pandemic period. A related limitation is the fact that adherence and participant responsiveness were assessed by the facilitators, who may introduce potential bias by overestimating their level of implementation and students’ behaviors. Thus, direct observations in classrooms through independent observers may be valuable in future studies. Despite the direction of the changes between pre- and post-intervention/follow-ups being in line with the expected results for the intervention group and not for the control group, the interaction effects between time and condition were not statistically significant, which precludes robust conclusions. Another limitation was that the program focused on teaching emotion awareness and self-management of emotions, which may not be fully captured by the self-report measures we used because they focus on the emotional climate in the classroom. A better emotional climate may arise from increased self-awareness and self-management of emotions, which were the focus of the intervention program, but these are not overlapping constructs. Future studies should also incorporate additional measures from multi-information sources (e.g., teachers, and parents) about children’s social–emotional skills. The program was implemented during a world health crisis (the COVID-19 pandemic) and some sessions were delivered online. Although the online delivery did not affect the attendance/dosage of the program, the threat to human life during that period made emotional management more difficult for everyone, which may have played against the utility of the program in bringing about change. Indeed, mental health difficulties in children (e.g., depression, anxiety, PTSD) increased during the COVID-19 lockdown (Panchal et al., 2021). Additionally, this fact may highlight the importance of promoting social–emotional skills in person in the classroom, as a context for not only modeling those skills but also for improving, *in loco,* social connectedness, and cooperation.

## 5. Conclusion

Taken together, results from the current study point to the feasibility and, to some extent, the efficacy of a compassion-based socioemotional skills program on fostering children’s empathy and soothing and vitality emotions in the school context. These findings concur with the possibility of kindness and compassion being caught, taught, and cultivated in the school context, irrespective of people’s age and cultural background (Schonert-Reichl et al., 2012; Coles, 2015; Cayton, 2017). When empathy and compassion attitudes and actions are incorporated into the education system (including students, teachers, staff, and school culture) a dynamic process enfolds that enhances altruism, cohesion, cooperation, and compassion in societies, with benefits for all human beings (Coles, 2015). Additionally, this kind of universal action may promote resilient, healthy, and sustainable human societies, which is aligned with the international guidelines for Sustainable Development Goals (OECD, 2021a,b). As such, it seems warranted that continuous work is devoted to investigating how to better promote these socioemotional regulation skills effectively and from an early age, as was intended by The Me and the Us of Emotions.

## Data availability statement

The raw data supporting the conclusions of this article will be made available by the authors, without undue reservation.

## Ethics statement

The studies involving humans were approved by Ethical Committee for Health of the Universidade Portucalense Infante D. Henrique, Porto, Portugal. The studies were conducted in accordance with the local legislation and institutional requirements. Written informed consent for participation in this study was provided by the participants’ legal guardians/next of kin.

## Author contributions

AX, PV, LP, PM, BP, and SM: study conception and design. SM and MT: acquisition of data. AX, PV, and BP: analysis of data. AX, PV, and LP: interpretation of data and drafting of manuscript. All authors contributed to the article and approved the submitted version.

## Footnotes

^1^ https://emocoes.isr.uc.pt

## References

[ref1] Albuquerque I. Matos M. Cunha M. Galhardo A. Palmeira L. Lima M. (2019). Emotional Climate in the Classroom Scale - Children (ECCS - C). Unpublished Instrument. University of Coimbra, Portugal.

[ref2] Aronen E. T. Vuontela V. Steenari M. R. Salmi J. Carlson S. (2005). Working memory, psychiatric symptoms, and academic performance at school. Neurobiol. Learn. Mem. 83, 33–42. doi: 10.1016/j.nlm.2004.06.010 15607686

[ref3] Berg J. Osher D. Moroney D. Yoder N. (2017). The Intersection of School Climate and Social and Emotional Development. American Institutes for Research. Available at: https://www.air.org/sites/default/files/downloads/report/Intersection-School-Climate-and-Social-and-Emotional-Development-February-2017.pdf

[ref4] Bluth K. (2017). The Self-Compassion Workbook for Teens: Mindfulness and Compassion Skills to Overcome Self-Criticism and Embrace Who You Are. Oakland, CA: New Harbinger Publications.

[ref5] Bluth K. Gaylord S. A. Campo R. A. Mullarkey M. C. Hobbs L. (2016). Making friends with yourself: a mixed methods pilot study of a mindful self-compassion program for adolescents. Mindfulness 7, 479–492. doi: 10.1007/s12671-015-0476-6, PMID: 27110301 PMC4838201

[ref6] Calouste Gulbenkian Foundation. (2023). Gulbenkian Programme for Knowledge. Available at: https://gulbenkian.pt/en/initiatives/knowledge-programme/

[ref7] Carroll C. Patterson M. Wood S. Booth A. Rick J. Balain S. (2007). A conceptual framework for implementation fidelity. Implement. Sci. 2, 1–9. doi: 10.1186/1748-5908-2-40, PMID: 18053122 PMC2213686

[ref8] Cayton P. (2017). Compassion in Education: An Introduction to Creating Compassionate Cultures. London: Foundation for Developing Compassion and Wisdom.

[ref9] Cheang R. Gillions A. Sparkes E. (2019). Do mindfulness-based interventions increase empathy and compassion in children and adolescents: a systematic review. J. Child Fam. Stud. 28, 1765–1779. doi: 10.1007/s10826-019-01413-9

[ref10] Chierchia G. Singer T. (2016). “The neuroscience of compassion and empathy and their link to prosocial motivation and behavior” in Decision Neuroscience: An Integrative Perspective. eds. Dreher J. Tremblay L. (London: Elsevier Academic Press), 247–257. doi: 10.1016/B978-0-12-805308-9.00020-8

[ref11] Cipriano C. Naples L. H. Zieher A. Durlak J. Eveleigh A. Funero M.. (2021). The State of Evidence for Social and Emotional Learning: A Contemporary Meta-Analysis of Universal School-Based SEL Interventions. Child Development. Available at: https://osf.io/r246m 10.1111/cdev.13968 37448158

[ref12] Clarke A. M. Morreale S. Field C. A. Hussein Y. Barry M. M. (2015). What Works in Enhancing Social and Emotional Skills Development During Childhood and Adolescence? A Review of the Evidence on the Effectiveness of School-Based and Out-of-School Programmes in the UK. Retrieved from the U.K. Health Promotion Research Centre Website. Available at: https://www.gov.uk/government/uploads/system/uploads/attachment_data/file/411492/What_works_in_enhancing_social_and_emotional_skills_development_during_childhood_and_adolescence.pdf.

[ref13] Cohen J. (1992). Statistical power analysis. Curr. Dir. Psychol. Sci. 1, 98–101. doi: 10.1111/1467-8721.ep10768783

[ref14] Coles M. I. (Ed.) (2015). Towards the Compassionate School: From Golden Rule to Golden Thread. London: Institute of Education Press.

[ref15] Collaborative for Academic, Social and Emotional Learning (2015). CASEL Guide: Effective Social and Emotional Learning Programs - Middle and High School Edition. Available at: https://pg.casel.org

[ref16] Corcoran R. P. Cheung A. C. Kim E. Xie C. (2018). Effective universal school-based social and emotional learning programs for improving academic achievement: a systematic review and meta-analysis of 50 years of research. Educ. Res. Rev. 25, 56–72. doi: 10.1016/j.edurev.2017.12.001

[ref17] Crean H. F. Johnson D. B. (2013). Promoting alternative thinking strategies (PATHS) and elementary school aged Children’s aggression: results from a cluster randomized trial. Am. J. Community Psychol. 52, 56–72. doi: 10.1007/s10464-013-9576-4, PMID: 23625456

[ref18] Denham S. A. Wyatt T. M. Bassett H. H. Echeverria D. Knox S. S. (2009). Assessing social-emotional development in children from a longitudinal perspective. J. Epidemiol. Community Health 63, i37–i52. doi: 10.1136/jech.2007.070797, PMID: 19098138

[ref19] Domitrovich C. E. Durlak J. A. Staley K. C. Weissberg R. P. (2017). Social-emotional competence: an essential factor for promoting positive adjustment and reducing risk in school children. Child Dev. 88, 408–416. doi: 10.1111/cdev.12739, PMID: 28213889

[ref20] Durlak J. A. (2016). Programme implementation in social and emotional learning: basic issues and research findings. Camb. J. Educ. 46, 333–345. doi: 10.1080/0305764X.2016.1142504

[ref21] Durlak J. A. Weissberg R. P. Dymnicki A. B. Taylor R. D. Schellinger K. B. (2011). The impact of enhancing students’ social and emotional learning: a meta-analysis of school-based universal interventions. Child Dev. 82, 405–432. doi: 10.1111/j.1467-8624.2010.01564.x, PMID: 21291449

[ref22] Elias M. J. Brackett M. A. Miller R. Jones S. Kahn J. Mahoney J. L.. (2019). “Developing social and emotional skills and attitudes and ecological assets” in Keeping Students Safe and Helping them Thrive: A Collaborative Handbook on School Safety, Mental Health, and Wellness. eds. Osher D. Mayer M. J. Jagers R. J. Kendziora K. Wood L. (Praeger/ABC-CLIO: Santa Barbara, CA), 185–209.

[ref23] Field A. (2013). Discovering Statistics Using IBM SPSS Statistics. London: Sage.

[ref24] Gadke D. L. Kratochwill T. R. Gettinger M. (2021). Incorporating feasibility protocols in intervention research. J. Sch. Psychol. 84, 1–18. doi: 10.1016/j.jsp.2020.11.004 33581765

[ref25] Gilbert P. (2005). “Compassion and cruelty: a biopsychosocial approach” in Conceptualisations, Research and Use in Psychotherapy. ed. Gilbert P. (London: Routledge), 9–74.

[ref26] Gilbert P. (2009). The Compassionate Mind: A New Approach to Life’s Challenges. Constable & Robinson, London.

[ref27] Gilbert P. (2014). The origins and nature of compassion focused therapy. Br. J. Clin. Psychol. 53, 6–41. doi: 10.1111/bjc.12043, PMID: 24588760

[ref28] Gilbert P. Choden K (2013). Mindful Compassion. Constable & Robinson, London, UK.

[ref29] Girard C. Ecalle J. Magnan A. (2013). Serious games as new educational tools: how effective are they? A meta-analysis of recent studies. J. Comput. Assist. Learn. 29, 207–219. doi: 10.1111/j.1365-2729.2012.00489.x

[ref30] Godinho S. (1988). Canção dos Abraços [Song]. On Canta com os amigos do Gaspar [Sing with the Gaspar’s Friends]. Portugal: Universal Music Portugal.

[ref31] Gonçalves D. R. O. (2019). Estudo Piloto de um Programa de Promoção de Competências Emocionais em Crianças do 3° ano de Escolaridade. [Pilot Study of a Social-Emotional Program for Children from 3rd Grade]. Master’s Thesis. Universidade Portucalense, Portugal.

[ref32] Gueldner B. A. Feuerborn L. L. Merrell K. W. (2020). Social and Emotional Learning in the Classroom: Promoting Mental Health and Academic Success. London: Guilford Publications.

[ref33] Henriques A. (2019). Avaliação do Clima Emocional na Sala de Aula: desenvolvimento e validação de um novo instrumento para adolescentes. [Assessment of the Emotional Climate in the Classroom: Development and Validation of a New Scale for Adolescents]. Master’s Thesis. Instituto Superior Miguel Torga, Coimbra, Portugal.

[ref34] Hutcherson C. A. Seppala E. M. Gross J. J. (2008). Loving-kindness meditation increases social connectedness. Emotion 8, 720–724. doi: 10.1037/a0013237, PMID: 18837623

[ref35] Jones S. M. Barnes S. P. Bailey R. Doolittle E. J. (2017). Promoting social and emotional competencies in elementary school. Futur. Child. 27, 49–72. doi: 10.1353/foc.2017.0003

[ref36] Karakasidou E. Raftopoulou G. Stalikas A. (2021). A self-compassion intervention program for children in Greece. Psychology 12, 1990–2008. doi: 10.4236/psych.2021.1212121

[ref37] Kirby J. N. Doty J. R. Petrocchi N. Gilbert P. (2017a). The current and future role of heart rate variability for assessing and training compassion. Front. Public Health 5:40. doi: 10.3389/fpubh.2017.00040, PMID: 28337432 PMC5340770

[ref38] Kirby J. N. Tellegen C. L. Steindl S. R. (2017b). A meta-analysis of compassion-based interventions: current state of knowledge and future directions. Behav. Ther. 48, 778–792. doi: 10.1016/j.beth.2017.06.003, PMID: 29029675

[ref39] Kirschner H. Kuyken W. Wright K. Roberts H. Brejcha C. Karl A. (2019). Soothing your heart and feeling connected: a new experimental paradigm to study the benefits of self-compassion. Clin. Psychol. Sci. 7, 545–565. doi: 10.1177/2167702618812438, PMID: 32655984 PMC7324152

[ref40] Klimecki O. M. Leiberg S. Ricard M. Singer T. (2014). Differential pattern of functional brain plasticity after compassion and empathy training. Soc. Cogn. Affect. Neurosci. 9, 873–879. doi: 10.1093/scan/nst060, PMID: 23576808 PMC4040103

[ref41] Kline R. B. (2015). Principles and Practice of Structural Equation Modeling. 4th Edn New York: Guilford Publications.

[ref42] Lamb R. L. Annetta L. Firestone J. Etopio E. (2018). A meta-analysis with examination of moderators of student cognition, affect, and learning outcomes while using serious educational games, serious games, and simulations. Comput. Hum. Behav. 80, 158–167. doi: 10.1016/j.chb.2017.10.040

[ref43] Lawson G. M. McKenzie M. E. Becker K. D. Selby L. Hoover S. A. (2019). The core components of evidence-based social emotional learning programs. Prev. Sci. 20, 457–467. doi: 10.1007/s11121-018-0953-y, PMID: 30443846 PMC6544145

[ref44] Mondi C. F. Giovanelli A. Reynolds A. J. (2021). Fostering socio-emotional learning through early childhood intervention. Int. J. Child Care Educ. Policy 15, 1–43. doi: 10.1186/s40723-021-00084-8 PMC11812064 39935621

[ref45] Neff K. D. Germer C. K. (2013). A pilot study and randomized controlled trial of the mindful self-compassion program. J. Clin. Psychol. 69, 28–44. doi: 10.1002/jclp.21923 23070875

[ref46] OECD (2019). Assessing students’ social and emotional skills through triangulation of assessment methods. OECD Educ. Work. Papers 208:6. doi: 10.1787/717ad7f2-en

[ref47] OECD (2021a). Positive, High-Achieving Students?: What Schools and Teachers Can Do. OECD Publishing, Paris.

[ref48] OECD (2021b). Beyond Academic Learning: First Results from the Survey of Social and Emotional Skills, OECD Publishing, Paris.

[ref49] Osher D. Kidron Y. DeCandia C. J. Kendziora K. Weissberg R. (2015). “Interventions to promote safe and supportive school climate” in Social Influences on Social-Emotional, Motivation, and Cognitive Outcomes in School Contexts. eds. Wentzel K. Ramani G. (New York, NY: Taylor Francis).

[ref50] Panchal U. Salazar de Pablo G. Franco M. Moreno C. Parellada M. Arango C.. (2021). The impact of COVID-19 lockdown on child and adolescent mental health: systematic review. Eur. Child Adolesc. Psychiatry 32, 1151–1177. doi: 10.1007/s00787-021-01856-w, PMID: 34406494 PMC8371430

[ref51] Schonert-Reichl K. A. Oberle E. Lawlor M. S. Abbott D. Thomson K. Oberlander T. F.. (2015). Enhancing cognitive and social–emotional development through a simple-to-administer mindfulness-based school program for elementary school children: a randomized controlled trial. Dev. Psychol. 51, 52–66. doi: 10.1037/a0038454, PMID: 25546595 PMC4323355

[ref52] Schonert-Reichl K. A. Smith V. Zaidman-Zait A. Hertzman C. (2012). Promoting children’s prosocial behaviors in school: impact of the “roots of empathy” program on the social and emotional competence of school-aged children. Sch. Ment. Heal. 4, 1–21. doi: 10.1007/s12310-011-9064-7

[ref53] Schonfeld D. J. Adams R. E. Fredstrom B. K. Weissberg R. P. Gilman R. Voyce C.. (2015). Cluster-randomized trial demonstrating impact on academic achievement of elementary social-emotional learning. Sch. Psychol. Q. 30, 406–420. doi: 10.1037/spq0000099, PMID: 25485463

[ref54] Singer T. Steinbeis N. (2009). Differential roles of fairness-and compassion-based motivations for cooperation, defection, and punishment. Ann. N. Y. Acad. Sci. 1167, 41–50. doi: 10.1111/j.1749-6632.2009.04733.x, PMID: 19580551

[ref55] Sklad M. Diekstra R. Ritter M. D. Ben J. Gravesteijn C. (2012). Effectiveness of school-based universal social, emotional, and behavioral programs: do they enhance students’ development in the area of skill, behavior, and adjustment? Psychol. Sch. 49, 892–909. doi: 10.1002/pits.21641

[ref56] Szalavitz M. Perry B. D. (2010). Born for Love: Why Empathy Is Essential--and Endangered. New York: William Morrow.

[ref57] Taylor R. D. Oberle E. Durlak J. A. Weissberg R. P. (2017). Promoting positive youth development through school-based social and emotional learning interventions: a meta-analysis of follow-up effects. Child Dev. 88, 1156–1171. doi: 10.1111/cdev.12864, PMID: 28685826

[ref58] Vroom E. B. Massey O. T. Yampolskaya S. Levin B. L. (2019). The impact of implementation Fidelity on student outcomes in the life skills training program. Sch. Ment. Health. 12, 113–123. doi: 10.1007/s12310-019-09333-1

[ref59] Webster-Stratton C. (1999). How to Promote Children's Social and Emotional Competence. United States: Sage.

[ref60] Weissberg R. P. Durlak J. A. Domitrovich C. E. Gullotta T. P. (2015). “Social and emotional learning: past, present, and future” in Handbook of Social and Emotional Learning: Research and Practice. eds. Durlak J. A. Domitrovich C. E. Weissberg R. P. Gullotta T. P. (New York: The Guilford Press), 3–19.

[ref61] Welford M. Langmead K. (2015). Compassion-based initiatives in educational settings. Educ. Child Psychol. 32, 71–80. doi: 10.53841/bpsecp.2015.32.1.71

[ref62] Xavier A. Vagos P. Palmeira L. Menezes P. Patrão B. Pereira S.. (2022). Children’s perspectives on using serious games as a complement to promoting their social-emotional skills. Int. J. Environ. Res. Public Health 19:9613. doi: 10.3390/ijerph19159613, PMID: 35954968 PMC9367770

[ref63] Zheng L. R. Oberle C. M. Hawkes-Robinson W. A. Daniau S. (2021). Serious games as a complementary tool for social skill development in young people: a systematic review of the literature. Simul. Gaming 52, 686–714. doi: 10.1177/10468781211031283

[ref64] Zhonggen Y. (2019). A meta-analysis of use of serious games in education over a decade. Int. J. Comput. Games Technol. 2019, 1–8. doi: 10.1155/2019/4797032

